# The Effect of Antimicrobial Photodynamic Inactivation on the Protein Profile of Dormant *Mycolicibacterium smegmatis* Containing Endogenous Porphyrins

**DOI:** 10.3390/ijms241813968

**Published:** 2023-09-12

**Authors:** Denis M. Shashin, Galina R. Demina, Irina A. Linge, Galina N. Vostroknutova, Arseny S. Kaprelyants, Alexander P. Savitsky, Margarita O. Shleeva

**Affiliations:** 1 A.N. Bach Institute of Biochemistry, Federal Research Centre ‘Fundamentals of Biotechnology’ of the Russian Academy of Sciences, Moscow 119071, Russia; dmshashin@gmail.com (D.M.S.); galinademina@yandex.ru (G.R.D.); vostroknutova@mail.ru (G.N.V.); arseny48@mail.ru (A.S.K.); 2 Central Institute for Tuberculosis, Moscow 107564, Russia; iralinge@gmail.com

**Keywords:** *Mycobacterium smegmatis*, porphyrin, photodynamic inactivation, dormant mycobacteria

## Abstract

During transition into a dormant state, *Mycolicibacterium* (*Mycobacterium*) *smegmatis* cells are able to accumulate free porphyrins that makes them sensitive to photodynamic inactivation (PDI). The formation of dormant cells in a liquid medium with an increased concentration of magnesium (up to 25 mM) and zinc (up to 62 µM) resulted in an increase in the total amount of endogenous porphyrins in dormant *M. smegmatis* cells and their photosensitivity, especially for bacteria phagocytosed by macrophages. To gain insight into possible targets for PDI in bacterial dormant mycobacterial cells, a proteomic profiling with SDS gel electrophoresis and mass spectrometry analysis were conducted. Illumination of dormant forms of *M. smegmatis* resulted in the disappearance of proteins in the separating SDS gel. Dormant cells obtained under an elevated concentration of metal ions were more sensitive to PDI. Differential analysis of proteins with their identification with MALDI-TOF revealed that 45.2% and 63.9% of individual proteins disappeared from the separating gel after illumination for 5 and 15 min, respectively. Light-sensitive proteins include enzymes belonging to the glycolytic pathway, TCA cycle, pentose phosphate pathway, oxidative phosphorylation and energy production. Several proteins involved in protecting against oxygen stress and protein aggregation were found to be sensitive to light. This makes dormant cells highly vulnerable to harmful factors during a long stay in a non-replicative state. PDI caused inhibition of the respiratory chain activity and destroyed enzymes involved in the synthesis of proteins and nucleic acids, the processes which are necessary for dormant cell reactivation and their transition to multiplying bacteria. Because of such multiple targeting, PDI action via endogenous porphyrins could be considered as an effective approach for killing dormant bacteria and a perspective to inactivate dormant mycobacteria and combat the latent form of mycobacteriosis, first of all, with surface localization.

## 1. Introduction

The causative agent of tuberculosis, *Mycobacterium tuberculosis*, is able to go into a dormant state [1], leading to the development of a latent form of the disease. In this form, mycobacteria persist in the human body for a long time and, under the influence of a number of factors (diabetes [2], human immunodeficiency virus, organ transplantation, tumor necrosis factor-alpha blockers, kidney dialysis [3], COVID-19 [4]), are able to proceed to multiply and cause the development of an active form of the disease. The situation with this disease is complicated by a high and growing level of antibiotic-resistant strains of *M. tuberculosis* and multidrug-resistant tuberculosis (MDR-TB), which are resistant to first-line antibiotics (isoniazid and rifampicin). According to the Centers for Disease Control and Prevention (https://www.cdc.gov/tb/ accessed on 1 June 2023), more than 2.6 million people could die each year from MDR-TB by 2050 if the spread of resistant strains is not stopped. The spread of extensive and multidrug-resistant strains of mycobacteria that cause severe infectious diseases dictates the need to develop new approaches to combat these diseases.

Previously, it was found that dormant forms of *Mycolicibacterium* (*Mycobacterium*) *smegmatis* (*Msm*) (a close genetic relative of *M. tuberculosis*) accumulate intermediate metabolites of heme synthesis (porphyrins) in a significant amount [5]. These molecules are photosensitizers capable of causing photodynamic inactivation (PDI) of this bacterial species [6] that can serve as a basis for the photodynamic therapy of tuberculosis. However, when conducting PDI of these cultures, it was found that not all bacteria in the population are equally sensitive to the action of light [6]. This may be due to the insufficient activity of porphyrin synthesis enzymes, which hinders the accumulation of endogenous heme synthesis intermediates, even in the presence of excess 5-aminolevulinic acid (ALA). In general, the study of factors which lead to an increase in the concentration of endogenous porphyrins in pathogenic bacteria is a promising direction, since, in this case, the problem of the toxic effect of a photosensitizer (PS) on mammalian cells, which is observed when using exogenous PS [7], is solved, as well as the problem of targeted delivery of these substances to bacterial cells. Studies have also reported the selective inactivation of bacteria without causing significant harm to human tissue [8]. It seems promising to study in detail the possibility of modulating the activity of porphyrin synthesis enzymes through various chemical and physical factors, which can subsequently be applied under the conditions of the host organism. 

It is known that divalent metal ions can stimulate the activity of heme synthesis enzymes. The enzyme porphobilinogen synthase (aminolevulinate dehydratase) from *E. coli*, which catalyzes the asymmetric condensation and cyclization of two molecules of 5-aminolevulinate, which is the first general step in the biosynthesis of tetrapyrrole pigments, uses metal ions (Zn^2+^, Mg^2+^, K^+^ and Na^+^) as cofactors that are located in several places, including the active site and allosteric sites [9]. The active center of this enzyme contains Zn^2+^. It has been demonstrated that this enzyme is allosteric in heme synthesis, and its activity in *E. coli* cells increases in the presence of magnesium ions [9]. In the present study, the effect of zinc and magnesium ions on photosensitivity and stimulation of the accumulation of endogenous porphyrins in *M. smegmatis* cells was investigated in order to obtain dormant *Msm* cells with maximum light sensitivity. By using such cells, we aim to gain an insight into the mechanism of cell inactivation by PDI. Namely, by using the proteomic approach, we hope to characterize the changes in the proteomic profile after PDI in order to elucidate a protein cohort which has the most sensitivity to illumination.

## 2. Results

### 2.1. Influence of Metal Ions on the Accumulation of Porphyrins in Dormant Forms of M. smegmatis and Their Photosensitivity

The potential impact of metal ion concentrations in the growth medium on the photosensitivity of dormant *M. smegmatis* cells was studied by examining bacterial cells engulfed by macrophages, which are the primary host cells where pathogenic mycobacteria accumulate. 

**Figure 1:**
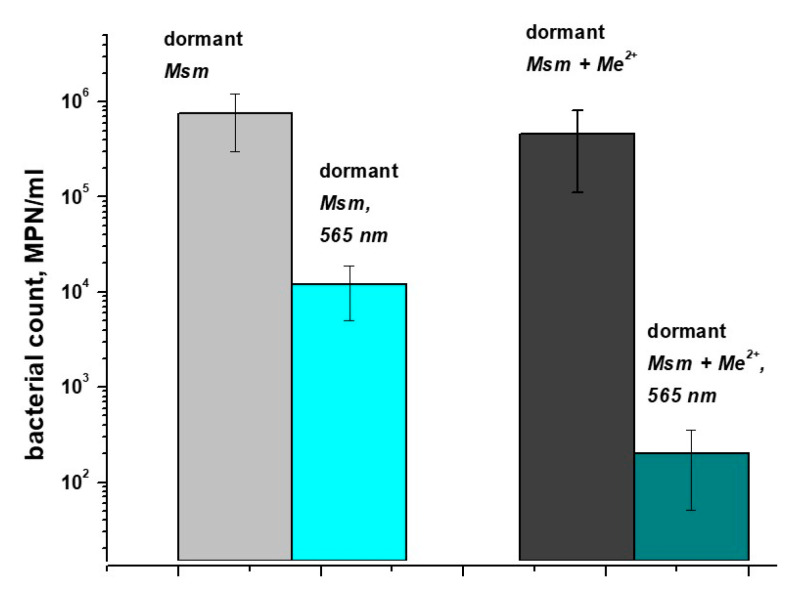
Survival of dormant *M. smegmatis* cells in macrophages under illumination. Dormant *Msm* cells obtained under standard (D_st_) and elevated (D_Me_) concentrations of Mg^2+^ and Zn^2+^ were added to peritoneal macrophages and incubated for 16 h. After washing macrophages with captured *Msm*, they were illuminated with LED 565 nm for 30 min at a power density of 180 mW. After illumination, macrophages were lysed and the concentration of viable bacteria was estimated with an MPN assay. This experiment was repeated twice. Bars represent SD.

The viability of dormant bacterial cells in macrophages decreased after exposure to light with a wavelength of 565 nm (corresponding to the one Q-band of porphyrin absorption). Light with longer wavelengths, such as 565 nm, can penetrate deeper into biological tissues compared to shorter wavelengths, such as 400 nm. However, in contrast to in vitro cultures [6], the difference in the number of viable bacteria between illuminated and unilluminated samples was less pronounced for bacteria inside macrophages (Figure 1). Thus, when the culture was illuminated in vitro in liquid medium, the viability of dormant mycobacteria decreased from 1.2 × 10^8^ ± 7 × 10^7^ to 2 × 10^3^ ± 9 × 10^2^ cells per mL. When macrophages phagocytosing these bacteria were illuminated, the viability of bacterial cells decreased from 8 × 10^5^ ± 4.3 × 10^5^ to 9 × 10^3^ ± 4.2 × 10^3^ cells per ml of culture. Obviously, this difference of ~1000 times could be explained by a different amount of accumulated porphyrins in both cases. In order to improve the photodynamic effect for mycobacteria captured by macrophages, the increase in the amounts of endogenous photosensitizers in bacterial cells looks promising.

**Figure 2:**
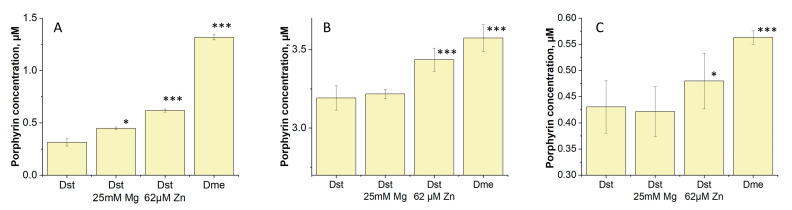
Amount of porphyrins extracted from dormant *M. smegmatis* cells. Dormant *Msm* cells obtained under standard (D_st_) and elevated (D_Me_) concentrations of Mg^2+^ and Zn^2+^ were extracted with chloroform–methanol and 2% triton X-100X100. Porphyrin concentration in supernatant (**A**), chloroform–methanol (**B**) and 2% triton X-100 (**C**). Asterisks indicate that the results are significantly different from the control (D_st_) according to Student’s *t*-test. Student’s test assuming unequal variance was performed for estimation of significance for comparative data. P-values are indicated as follows: * = *p* < 0.05, *** = *p* < 0.001.

Because the activity of some enzymes of porphyrin synthesis strongly depends on metallic ions [9,10], we checked how various concentrations of metal ions Mg^2+^ and Zn^2+^ added into the media influence porphyrin synthesis in an axenic culture of *Msm* and in cells captured by macrophages. To extract the accumulated porphyrins from the cells, a sequential extraction method was employed. At first, a chloroform–methanol–water solvent system was used, followed by extraction with PBS-Triton. The choice of extraction systems was made because the dominant porphyrin in dormant mycobacteria is the hydrophobic tetramethyl ester of coproporphyrin, which is extracted with organic solvents [5]. We observed that, with an increase in the concentration of magnesium by 5 times (from 5 mM to 25 mM) and with an increase in the concentration of zinc by 10 times (from 6.2 µM to 62 µM) relative to the standard Sauton medium [11], there was an increase in the production of endogenous porphyrins in dormant forms of *Msm* in the last case (Figure 2A–C). At the same time, a synergetic effect of two ions resulted in the highest production of porphyrins in these cultures (Figure 2A–C).

**Figure 3:**
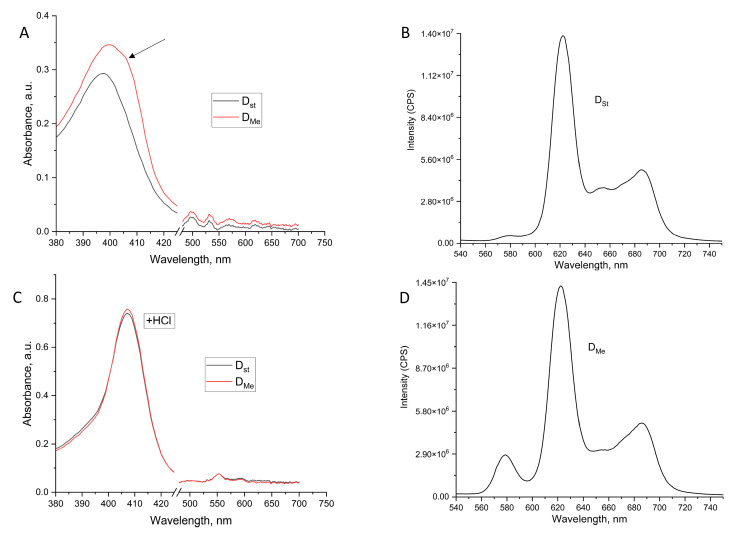
Absorption and fluorescence spectra of chloroform–methanol extract of dormant *M. smegmatis* cells. Dormant *Msm* cells were obtained under standard (D_st_) and elevated (D_Me_) concentrations of Mg^2+^ and Zn^2+^ as described in the Methods. Absorption spectra of extracts (**A**) and acidificated extract (**B**). Fluorescent spectra (excitation 400 nm) of extract from D_st_ cells (**C**) and D_Me_ cells (**D**). The arrow indicates a shoulder appeared in the Soret band.

In the absorption spectra of the chloroform–methanol extract of the dormant *Msm* cells grown in the presence of the elevated concentration of metal ions, a noticeable shoulder appears in the Soret band in the long-wavelength region (Figure 3A), which can be interpreted as the appearance of a metalloporphyrin, most likely Zn-porphyrin.

This assumption is supported by two facts. First, in 0.1 N HCl, after a few hours, the spectra of the extracts obtained under standard conditions and the extract of cells obtained at an increased concentration of metal ions became identical (Figure 3B), which can be explained by the fact that the Zn ions in acidic solutions easily dissociate from the porphyrin macrocycle [12]. Secondly, in contrast to dormant cells obtained under standard conditions (Figure 3C), the fluorescence spectrum of the extract obtained from cells grown under elevated concentration of metal ions corresponded to the fluorescence of the mixture of free porphyrin (maxima 620 and 685 nm) and Zn-porphyrin (the main maximum of 580 nm) (Figure 3D).

**Figure 4:**
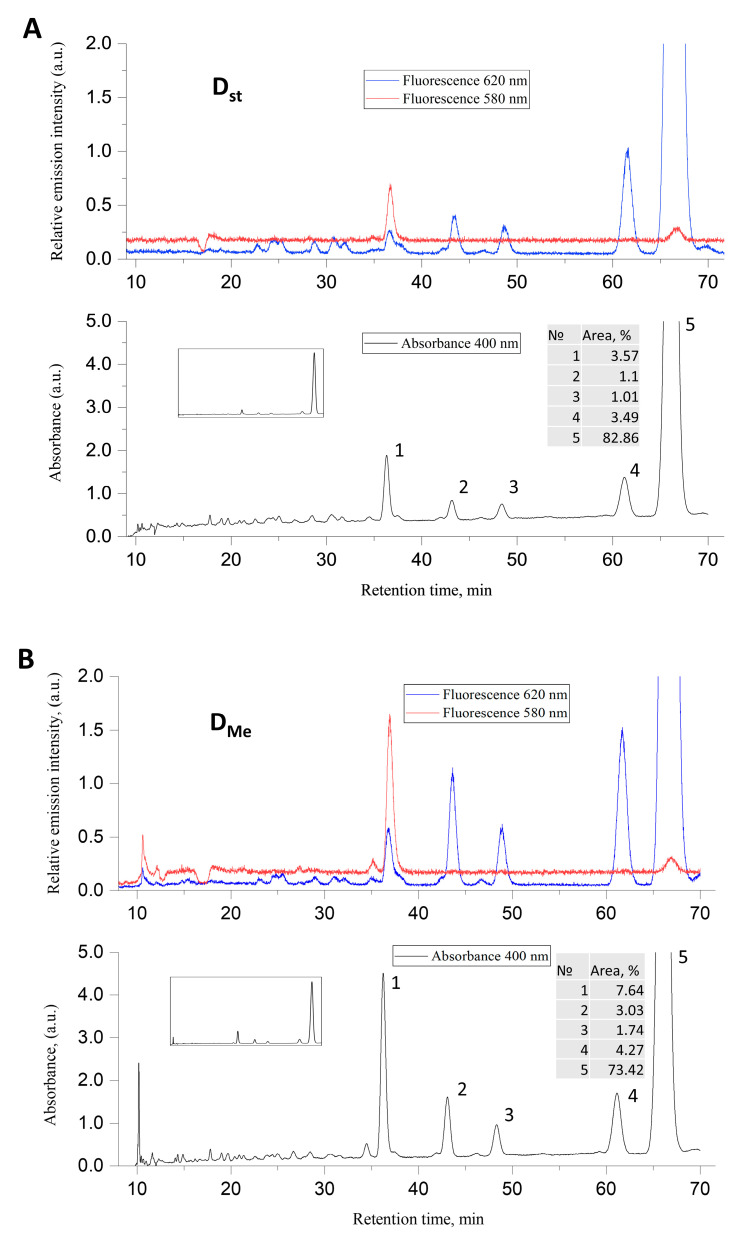
HPLC analysis chloroform–methanol extract of dormant *M. smegmatis* cells. Dormant *Msm* cells were obtained under (**A**) standard (D_st_) and (**B**) elevated (D_Me_) concentrations of Mg^2+^ and Zn^2+^ as described in the Methods. A C18 Tosoh Bioscience column was used; elution rate 1 mL min^−1^ and injection sample size 20 μL, gradient mode and gradient program as described in the Methods. The spectrofluorometer was set at 400 nm for absorption, fluorescence excitation wavelength was 400 nm and fluorescence emissions were detected at wavelengths of 580 and 620 nm. The numbers point to peaks observed at 400 nm.

HPLC analysis of porphyrins extracted from dormant mycobacteria grown at elevated concentrations of zinc and magnesium revealed five characteristic peaks (Figure 4). The peak corresponding to a retention time of 68 min corresponds to coproporphyrin tetramethyl ether. The peak with a retention time of 38 min and a fluorescence of 580 nm in the presence of metals increases by a factor of 2.1. The fluorescence at 580 nm indicates the presence of Zn—porphyrin [12]. Additionally, the peak with a retention time of 48 min increased by 3 times. The rest of the peaks changed insignificantly.

Accordingly, the illumination of macrophages containing dormant mycobacteria grown in the presence of an increased concentration of metals and containing increased amounts of endogenous porphyrins revealed a greater efficiency of photodynamic inactivation (Figure 1). Therefore, in the following experiments, two types of dormant cells with standard and elevated concentrations of metal ions were used.

### 2.2. Influence of Metals on the Representation of Proteins of Porphyrin Metabolism in Dormant M. smegmatis Cells

The observed increase in porphyrin levels in cells grown in the presence of higher metal ion concentrations can be attributed to the enhanced synthesis of enzymes involved in porphyrin metabolism. In order to check this possibility, one-dimensional electrophoresis was performed in 12% PAAG with SDS-Na for dormant cells obtained under standard (D_st_) and elevated concentrations of metal ions (D_Me_). Each protein band excised from the gel of D_st_ and D_Me_ samples was analyzed using MALDI TOF. In the presence of metals, there is a zone containing glutamate-1-semialdehyde-2,1-aminomutase/*MSMEG_0969*, hemL (involved in porphyrin biosynthesis by the C5 pathway at the second step), in contrast to the corresponding zone with the same mobility in the protein sample obtained from D_st_ cells, in which this enzyme was not detected with MALDI TOF. Evidently, the concentration of MSMEG_0969 protein increased in the D_Me_ sample with a high metal concentration (Tables S1 and S2). As a result of the activity of this protein, the formation of 5-aminolevulinic acid should occur, because this protein is rate-limiting in heme biosynthesis [13]. At the same time, the zone containing coproheme decarboxylase/*MSMEG_2782*, chdC—(ChdCs) that catalyzes the final step in heme b biosynthesis [13] was found in D_st_ cells, but the corresponding zone in the sample obtained from D_Me_ cells was not found (Tables S1 and S2). This might result in the elevation of intermediate substances in this pathway, including porphyrins.

Interestingly, the protein zone containing the copper/zinc superoxide dismutase/*MSMEG_0835* in D_st_ cells did not contain this enzyme in the D_Me_ cells. This enzyme destroys radicals which are normally produced within the cells and are toxic to biological systems. This fact weakens the protective properties of these mycobacteria and makes them more vulnerable to light exposure.

### 2.3. Primary Protein Targets for Photodynamic Inactivation of M. smegmatis

**Figure 5:**
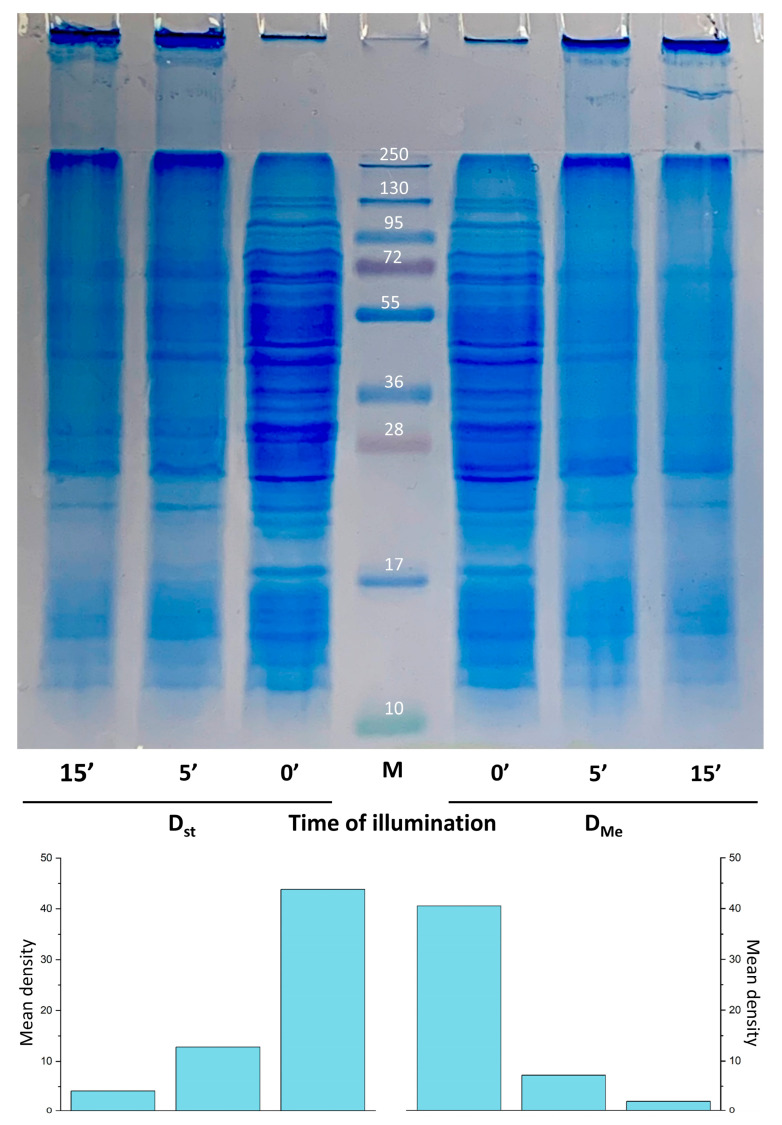
SDS electrophoresis of proteins obtained from dormant *M. smegmatis* cells before and after illumination with LED 565 nm. Dormant *Msm* cells obtained under standard (D_st_) and an elevated (D_Me_) concentration of Mg^2+^ and Zn^2+^ were subjected to LED 565 nm illumination for 5- and 15-min. Total cell proteins were extracted by 2% SDS and used for SDS gel electrophoresis followed by Coomassie staining. Concentration of proteins for each sample was identical (70 mg). 0′—corresponds to unilluminated samples. M—protein standards. Diagrams below represent mean density of protein bands between 130 kDa and 10 kDa for each protein track. Mean density—mean grey value of pixels extracted from monochrome image of electrophoresis gel with background correction.

We illuminated dormant forms of *M. smegmatis* (D_st_ and D_Me_) for 5, 15 and 30 min at a power density of 180 mW/cm^2^. After that, proteins were extracted and equal amounts of protein (70 mg) of illuminated and non-illuminated dormant *Msm* cells were separated using 1D SDS electrophoresis. Stained protein zones were excised and analyzed using MALDI-TOF. It was found that the higher the light dose, the less protein bands were observed in the separating gel and the more protein remained at the top of the concentrating gel and at the interface between the concentrating and separating gels (Figure 5). Evidently, this was caused by significant cross-linking of the proteins of illuminated cells via the generation of reactive oxygen species (ROS) [14].

We analyzed all visibly stained protein lines (bands) in a separating gel for samples before and after illumination for 5 and 15 min. Then, after MALDI TOF identification of the proteins in each band we compared the protein profile for each sample in order to find the presence or absence of corresponding protein in the sample after illumination (Tables S1 and S2).

**Figure 6:**
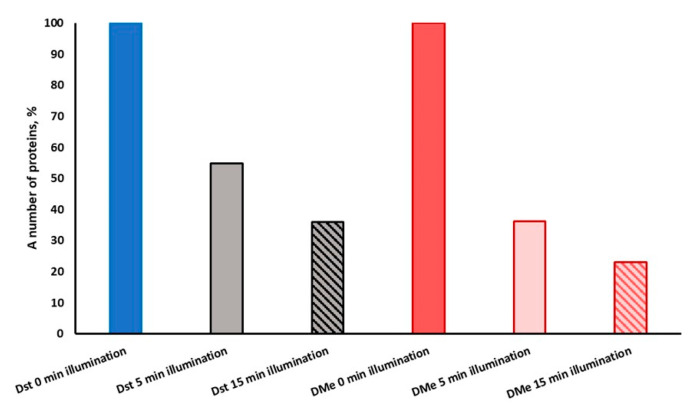
The number of proteins of dormant forms of *M. smegmatis* grown under standard conditions (D_st_) and at an elevated concentration of metal ions (D_Me_) which are stable to PDI for 5 min and 15 min of illumination. The number of stable proteins was calculated as a percentage of total number of protein visible in SDS gel (Figure 4). The 100% represents protein numbers in unilluminated samples.

It was found that 45.2% of individual proteins in D_st_ cells determined with MALDI TOF disappeared under illumination for 5 min (light dose 54 J/cm^2^). With an increase in the time of light exposure to 15 min (light dose 162 J/cm^2^), 63.9% of proteins disappeared from the separating gel. However, 54.8% and 36.1% of proteins remained stable under light exposure for 5 min and 15 min, respectively (Figure 6, Table S1). The same effect on proteins was observed in D_Me_ cells. In this case, the illumination led to disappearance by up to 63.7% for 5 min and 77% under illumination for 15 min. The percentage of stable proteins in D_Me_ cells after illumination was 36.3% (5 min) and 23% (15 min).

**Figure 7:**
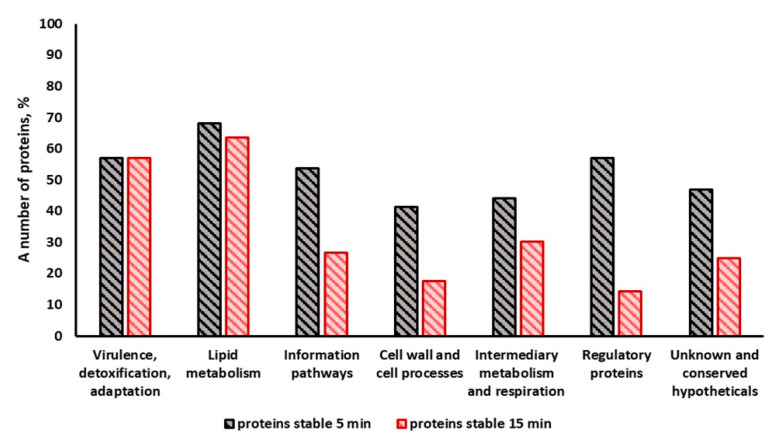
The stability of proteins in different functional categories of dormant forms of *M. smegmatis* under illumination for 5 min and 15 min. The number of stable proteins was calculated as a percentage of total number of visible proteins in SDS gel for every functional category (Figure 4). Protein number reached 100% in the unilluminated samples. The bacterial cells were grown under standard conditions.

When analyzing changes in the number of proteins within different functional categories, it was revealed that the greatest changes (up to extinction of the proteins) occurred in the categories “information pathways”, “cell wall and cell processes”, “intermediary metabolism and respiration” and “regulatory proteins” (Figure 7). For the number of proteins in the functional categories “virulence, detoxification, adaptation” and “lipid metabolism”, the decrease was not so significant (Figure 7). Most of the changes occurred with a short light exposure for 5 min (light dose 54 J/cm^2^). Increasing the light dose to 324 J/cm^2^ (30 min exposure) resulted in the almost complete disappearance of proteins in the area of the separating gel and in their accumulation in the concentrating gel, indicating a severe aggregation of the proteins. Under the influence of light, more than 33 proteins essential for vitality were damaged (Tables S1 and S2).

Most of the proteins of the dormant *Msm* forms which disappeared during exposure to light had membrane localization (Tables S1 and S2). A more detailed analysis of proteins disappearing from the separating gel revealed that they belonged to different metabolic pathways and reactions (see below).

#### 2.3.1. Enzymes of the Central Metabolism

**Figure 8:**
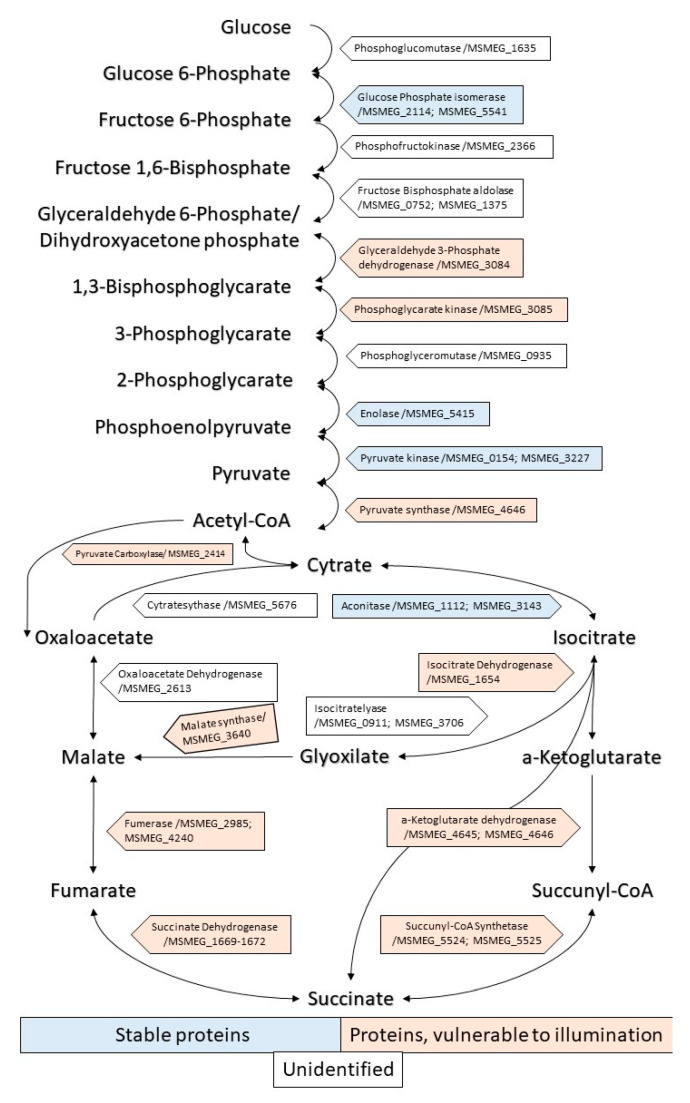
Influence of illumination with 565 nm on enzymes of the glycolytic pathway, citrate cycle and the glyoxylate shunt of dormant *M. smegmatis*.

After 5 min of illumination, a number of enzymes involved in the glycolysis pathway disappeared as individual proteins: glycerol-3-phosphate dehydrogenase 2 (*MSMEG_6761*); glyceraldehyde-3-phosphate dehydrogenase (*MSMEG_3084*); pyruvate synthase (*MSMEG_4646*) and glycerol kinase/*MSMEG_6759* (glpK), which catalyzes the conversion of glycerol to glycerol 3-phosphate. Further exposure to light (15 min) led to the disappearance of phosphoglycerate kinase (*MSMEG_3085*) (Table S1, Figure 8).

Pyruvate carboxylase (*MSMEG_2412*), which catalyzes the conversion of pyruvate to oxaloacetate, was found to be sensitive to a small dose of light (Table S1, Figure 8). In addition, under 5 min of illumination, the enzymes of the pyruvate dehydrogenase complex disappeared, namely pyruvate dehydrogenase E1 component/*MSMEG_4323* (aceE) and 2-oxoglutarate dehydrogenase E1 component/*MSMEG_5049* (sucA). Dihydrolipoyl dehydrogenase/*MSMEG_0903* (lpdA) is involved in energy metabolism as a part of the E3 component of pyruvate dehydrogenase and the alpha-ketoacid dehydrogenase complex. It is also involved in antioxidant defense.

NAD-glutamate dehydrogenase/*MSMEG_4699* (gdh), which is involved in the utilization of glutamate generating 2-oxoglutarate from L-glutamate, was sensitive for 5 min of illumination.

Among the Krebs cycle, only two enzymes, aconitase (*MSMEG_3143*) and citrate synthase I (*MSMEG_5672*), were light-resistant. The majority of enzymes of the tricarboxylic acid cycle were found to be sensitive even to a small (5 min) light dose: isocitrate dehydrogenase (*MSMEG_1654*), a key enzyme in the tricarboxylic acid cycle; alfa-ketoglutarate dehydrogenase (*MSMEG_4645*); ADP-forming succinate-CoA ligase (*MSMEG_5525*); fumarate hydratase class II (*MSMEG_5240*); and succinate dehydrogenase (*MSMEG_1672*) (Table S1, Figure 8).

The important enzyme of the glyoxylate shunt also disappeared; malate synthase G/*MSMEG_3640* (glcB), involved in the glyoxylate bypass (second step), is an alternative to the tricarboxylic acid cycle.

Transketolase/*MSMEG_3103* (tkt), is the cytosolic enzyme of the pentose phosphate pathway, a major route for the biosynthesis of the pentose sugars deoxyribose and ribose was highly sensitive to illumination.

Among the light-sensitive enzymes, there are those involved in heme biosynthesis: glutamate-1-semialdehyde-2,1-aminomutase/*MSMEG_0969* (hemL), involved in porphyrin biosynthesis via the C5 pathway (at the second step), and coproheme decarboxylase/M*SMEG_2782* (chdC), which catalyzes the final step in heme b biosynthesis.

#### 2.3.2. Oxidoreductases

Cytochrome D ubiquinol oxidase subunit I (*MSMEG_3233*), involved in the respiratory chain (terminal step) when cells are grown at low aeration, disappeared after only 5 min of light exposure (Table 1). Despite subunit II of cytochrome D ubiquinol oxidase (*MSMEG_3232*) remaining stable for up to 15 min of exposure, the whole carrier evidently lost its functionality.

The beta subunit (*MSMEG_4936*) of ATP synthase F1-F0, which produces ATP from ADP in the presence of a proton gradient across the membrane, disappeared after 5 min. At the same time, the gamma subunit (*MSMEG_4937*) and epsilon subunit (*MSMEG_4935*) of ATP synthase F1-F0 remained stable (Table 1 and Table S1).

Two oxidoreductases (NAD(P)/FAD-dependent oxidoreductase/*MSMEG_1030* (5 min) and quinone oxidoreductase/*MSMEG_3106,* which catalyze the one electron reduction of certain quinones (15 min), were sensitive to illumination.

F420-dependent glucose-6-phosphate dehydrogenase/*MSMEG_0777* (fgd), which catalyzes the oxidation of glucose-6-phosphate to 6-phosphogluconolactone using the coenzyme F420 and alpha oxoglutarate ferredoxin oxidoreductase, and beta subunit/*MSMEG_4645* (orB) also disappeared after illumination (Table 1).

#### 2.3.3. Various Metabolic Enzymes (Table 2)

The following enzymes that participated in the molybdopterin biosynthesis were sensitive to 15 min of illumination: molybdopterin biosynthesis protein/*MSMEG_5485*, which catalyzes the dehydration of 4A-hydroxytetrahydropterins, and molybdenum cofactor synthesis domain protein/*MSMEG_5702* (sensitive to 5 min of illumination), which is involved in the biosynthesis of a demolybdo-cofactor (molybdopterin).

Bacterioferritin/*MSMEG_3564* (involved in iron storage) and the siderophore binding protein/*MSMEG_5191*, which helps the organism accumulate iron, disappeared with an increased light dose (15 min).

All the below-cited enzymes disappeared after 5 min of light exposure (Table 2 and Table S1): the proteins involved in the synthesis of pyridoxine and pyridoxal phosphate (*MSMEG_5136*, *MSMEG_5243*); orotate phosphoribosyltransferase/*MSMEG_6520*, which is involved in pyrimidine biosynthesis; anthranilate synthase component 2/*MSMEG_0029*—involved in the biosynthesis of tryptophan (at the first step); (3R)-hydroxyacyl-ACP dehydratase subunit HadC/*MSMEG_1342*, which is involved in fatty acid synthesis type II (fas-II); acyl-CoA synthetase/*MSMEG_3767* (involved in lipid biosynthesis and degradation); NAD/mycothiol-dependent formaldehyde dehydrogenase/*MSMEG_4340* (involved in the reduction of S-nitroglutathione); UTP--glucose-1-phosphate uridylyltransferase/*MSMEG_5471* (galU) (plays a role in stationary phase survival); phosphoribosylformylglycinamidine synthase II/*MSMEG_5824* (purl) (involved in de novo purine biosynthesis).

Trehalose-6-phosphate synthase/*MSMEG_5892* (otsA) is involved in osmoregulatory trehalose biosynthesis and participates in dormancy development and maintaining a dormant state [1].

Polyketide synthase Pks13/*MSMEG_6392* is involved in the final steps of mycolic acid biosynthesis and catalyzes the condensation of two fatty acyl chains [15].

#### 2.3.4. Biopolymer Synthesis Enzymes

Among the enzymes participating in biopolymer synthesis, DNA polymerase III, beta subunit/*MSMEG_0001* (dnaN) was found to be sensitive to illumination.

Proteins involved in transcription were damaged under 5 min light exposure: DNA-directed RNA polymerase, beta subunit/*MSMEG_1367* (rpoB) and DNA-directed RNA polymerase, beta’ subunit/*MSMEG_1368* (rpoC).

**Figure 9:**
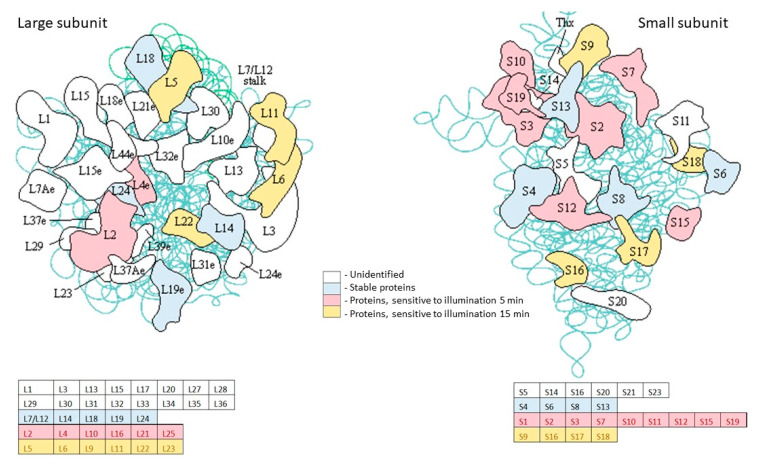
Influence of illumination with 565 nm on ribosomal proteins of dormant *M. smegmatis*. The scheme of ribosome was taken from KEGG.

Among the ribosomal proteins, a sufficiently large number were found to be sensitive to light exposure (Table S1, Figure 9). Thus, after 5 min of illumination, the following proteins disappeared: ribosomal protein S2/*MSMEG_2519* (rpsB); ribosomal protein S3/*MSMEG_1442* (rpsC); ribosomal protein L25, Ctc-form/*MSMEG_5431*; 50S ribosomal protein L16/*MSMEG_1443* (rplP); 30S ribosomal protein S11/*MSMEG_1522*, rpsK; 50S ribosomal protein L10/*MSMEG_1364*, rplJ; 30S ribosomal protein S7/*MSMEG_1399*, rpsG; 30S ribosomal protein S12/*MSMEG_1398*, rpsL; 30S ribosomal protein S15/*MSMEG_2654*, rpsO; 30S ribosomal protein S19/*MSMEG_1440*, rpsS; 30S ribosomal protein S10/*MSMEG_1435*, rpsJ; 50S ribosomal protein L21/*MSMEG_4625*,rplU; 30S ribosomal protein S1/*MSMEG_3833*, rpsA; 50S ribosomal protein L2/*MSMEG_1439*, rplB; 50S ribosomal protein L4*/MSMEG_1437*, rplD.

After 15 min of illumination, these additional ribosomal proteins disappeared: 50S ribosomal protein L5/*MSMEG_1467*, rplE; 50S ribosomal protein L6/*MSMEG_1470*, rplF; 30S ribosomal protein S16/*MSMEG_2435*, rpsP; 50S ribosomal protein L22/*MSMEG_1441*, rplV; 30S ribosomal protein S9/M*SMEG_1557*, rpsI; 50S ribosomal protein L20/M*SMEG_3791*, rplT; 30S ribosomal protein S17/M*SMEG_1445*, rpsQ; 50S ribosomal protein L23/M*SMEG_1438*, rplW; 30S ribosomal protein S18/M*SMEG_6895*, rpsR; 50S ribosomal protein L11/M*SMEG_1346*, rplK; 50S ribosomal protein L9/M*SMEG_6894*, rplI.

The ribosome recycling factor/M*SMEG_2541* (frr) disappeared after 5 min of exposure to light. Frr is responsible for the recycling of ribosomes from one round of translation to another [16].

Some enzymes participating in protein biosynthesis were found to be light-sensitive: glutamyl-tRNA synthetase/M*SMEG_2383* (gltX); threonyl-tRNA synthetase/M*SMEG_2931* (thrS); aspartyl-tRNA synthetase/M*SMEG_3003* (aspS); phenylalanyl-tRNA synthetase, beta subunit/M*SMEG_3777*; valyl-tRNA synthetase/M*SMEG_4630* (Table S1).

#### 2.3.5. Regulatory Proteins (Table 3)

Fifteen minutes of illumination resulted in the disappearance of the DNA-binding response regulator/M*SMEG_5488* (mprA), which is a regulatory part of a two-component system (MPRAB system), and the single-stranded DNA-binding protein/M*SMEG_6896* (ssb), which is essential for the replication of the chromosome. It is also involved in DNA recombination and repair.

Among the response regulators, DNA-binding response regulator ResD/M*SMEG_0994* (resD); response regulator/M*SMEG_3246* and DNA-binding response regulator RegX3/M*SMEG_0937* (regX3) were found to be sensitive to light.

The following regulatory proteins disappeared after light exposure: glycogen accumulation regulator GarA/M*SMEG_3647*, which is involved in the regulation of the glutamate metabolism; and the negative regulator of genetic competence ClpC/mecB/M*SMEG_6091* (clpC1) which hydrolyses proteins in the presence of ATP. ClpC1 may interact with a CLPP-like protease involved in the degradation of denatured proteins.

#### 2.3.6. Enzymes of Defense Systems

The following proteins that participate in defense mechanisms showed sensitivity to light illumination (Table 4):

Chaperonin GroEL/M*SMEG_0880* (groL1) belongs to the chaperonin family of molecular chaperones and is required for the proper folding of many proteins.

- Trigger factor/M*SMEG_4674* (tig), which is involved in protein export. It acts as a chaperone by maintaining the newly synthesized protein in an open conformation.
- *MSMEG_3932*/alpha-crystallin stress protein induced by anoxia. It has a proposed role in the maintenance of long-term viability during dormancy and latent infections.
- Thioredoxin/M*SMEG_4917* participates in various redox reactions through the reversible oxidation of its active center dithiol to a disulfide, and dithiol–disulfide exchange reactions. Copper/zinc superoxide dismutase/M*SMEG_0835* (sodC), which destroys radicals which are normally produced within the cells and are toxic to biological systems. AhpC/TSA family protein/M*SMEG_4753*, which is a putative antioxidant protein.
- Glyoxalase family protein/M*SMEG_5680* which is important in the detoxification of methylglyoxal.

Under conditions of elevated concentrations of Mg and Zn ions, a more pronounced effect of illumination on the protein bands’ disappearance for both 5 and 15 min of illumination was registered (Table S2, Figure 5 and Figure 6). This effect can be explained by an elevated intracellular concentration of porphyrins under a high metal ion concentration, as was found (Figure 2).

### 2.4. Enzymes Stable under Light Exposure

Proteins which demonstrated stability after 15 min of illumination were found to belong to different metabolic reactions and pathways, including those which were considered above.

They include enzymes of the central metabolism, regulatory proteins, lipid metabolism, and ribosomal proteins. A significant number of proteins included in the defense system were also light resistant. A list of resistant proteins is presented in Table S3.

### 2.5. Influence of Photodynamic Inactivation on DCPIP Reduction by M. smegmatis Cells

From the above experiment it can be seen that most of the identified light-sensitive targets are membrane proteins (Tables S1 and S2), which is probably due to the localization of the dominant porphyrin (tetramethyl ester of coproporphyrin) in the membrane fraction of dormant *M. smegmatis* cells [5]. Therefore, the activity of biochemical reactions linked to the activity of the membrane-bound enzymes should be especially sensitive to illumination. To verify this suggestion, we tested the activity of the respiratory chain in *Msm* cells. Enzymes of the respiratory chain are localized in the inner (cytoplasmic) membrane and their activity could be tested as possible membrane-bound targets of photodynamic inactivation. Because dormant *Msm* bacteria exhibit negligible respiratory activity [17], for these experiments we used a culture of *M. smegmatis* in a stationary phase of growth (72 h) in the presence of 3 mM 5-aminolevulinic acid (ALA), which stimulates the synthesis of porphyrin in *Msm* cells [6]. Thus, we obtained a culture with measurable activity of the respiratory chain containing free porphyrins in an amount close to dormant *Msm* cells [6].

**Figure 10:**
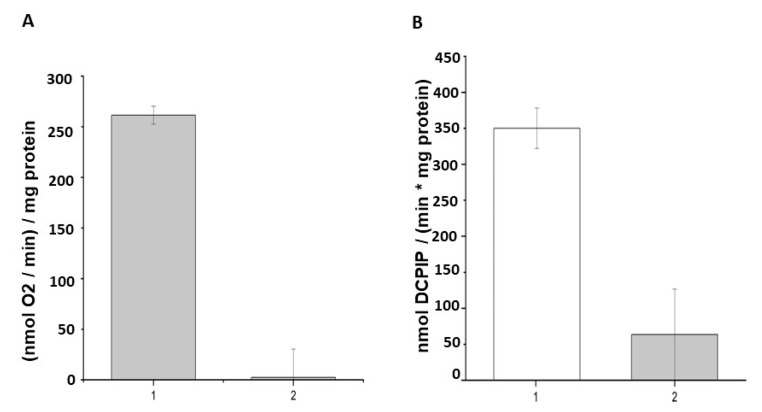
Influence of illumination with 565 nm on the respiratory chain activity of *M. smegmatis*. The 72-h-grown *Msm* culture in the presence 3 mM ALA was subject to illumination under 565 nm for 15 min. (**A**) The rate of total oxygen consumption and (**B**) the rate of reduction of the redox indicator 2,6-dichlorophenolindophenol (DCPIP). 1—control, 2—after illumination.

Activity of the respiratory chain for stationary phase *Msm* cells was assessed using the rate of total oxygen consumption (whole respiratory chain activity) and reduction of the redox indicator 2,6-dichlorophenolindophenol (DCPIP) (dehydrogenases-menaquinone mediated activity). It was found that, after 30 min of illumination of *Msm* cells grown in the presence of ALA, the activity of the respiratory chain substantially decreased (Figure 10) according to two experimental methods.).

## 3. Discussion

We have previously found that, during transition into a dormant state, *Mycobacterium smegmatis* is able to accumulate free porphyrins—namely, coproporphyrin III, uroporphyrin III, and their methyl esters endogenously [5]. This finding opens a possibility to inactivate dormant bacteria by PDI using endogenous PS [6], whilst exogenous PSs could not reach intracellular targets because of the highly impermeable cell wall of the dormant bacteria. In the present work, we used light with a 565 nm wavelength which can penetrate deeper into biological cultures compared to shorter wavelengths, such as 400 nm. The effectiveness of PDI obviously depends on the intracellular concentration of PS (porphyrins). The addition of 5-aminolevulinic acid (ALA) to the growth medium makes it possible to increase the concentration of endogenous porphyrins, which results in effective PDI for active forms of mycobacteria [6,18]. In the present study, we were able to modulate the concentration of free porphyrins in *Msm* cells by varying the concentration of Mg and Zn ions (Figure 2). According to protein profiling experiments, an elevated concentration of metal ions results in the up-regulation of synthesis of enzymes participating in the production of 5-aminolevulinic acid (Tables S1 and S2). At the same time, the amount of the terminal enzyme in synthesis of heme in mycobacteria—coproheme decarboxylase [19]—decreased (Tables S1 and S2). In summary, this should lead to an accumulation of intermediates in the heme synthesis pathway, which we observed experimentally for porphyrins. As a result, dormant cells showed higher sensitivity for PDI (Figure 1) when grown in the presence of an elevated concentration of metal ions. It is important that the effect of Zn plus Mg ions is noticeable in experiments with mycobacterial cells captured by macrophages that are closer to the situation in vivo. Retrospectively, an effect of synergetic action of metal ions could be used in practical application for the inactivation of a skin pathogen using PDI. 

There is a general opinion that the harmful effect of PDI on a cell is mediated by the production of a variety of reactive oxygen forms [14,20]. Less known is which targets in the cell are most sensitive for PDI and which mechanisms are responsible for the development of a bacteriocidic effect. According to a number of studies, performed using mainly exogeneous PSs lipids, proteins, DNA and membrane structures are considered as primary targets for PDI [20,21,22]. Recently, ferroptosis has been suggested as a possible mechanism of *Mycobacterium abscessus* death after ALA_PDI [23]. Bacterial lipids are altered during illumination in the presence of endogenous porphyrin. Thus, for *Staphylococcus aureus* containing intracellular coproporphyrin, it was shown that the amount of the lipid peroxidation product malondialdehyde increased, and intracellular potassium (K+) leakage was detected, indicating that the cell membrane became permeable upon light exposure [24].

Not discarding the significance of these structures in light sensitivity, some works indicate the importance of proteins (enzymes) as the main targets for PDI [21,25]. However, different bacterial species might have different primary targets for PDI [26]. Moreover, different targets could be primary for different PSs [27].

In the present study, we found that a significant number of proteins appeared in aggregates as a result of illumination (Figure 5). Similar protein aggregation has been found for *Staphylococcus aureus* after PDI using exogenous PS [28]. The mechanism of such aggregation is likely due to the cross-linking of proteins as a result of the carbonylation of amino acid residues due to ROS production [14,26] which should lead to functional damage of the protein.

It is interesting that, despite this study being the first investigation into the sensitivity to PDI of bacterial proteins in dormant cells, many vulnerable-to-light proteins are similar to those found to be sensitive to PDI for active bacteria and other types of PS. Thus, the analysis of protein profiling after PDI in the present study reveals that a significant number of enzymes belonging to the glycolytic pathway, TCA cycle and pentose phosphate pathway disappear from the pool of intact proteins. Similar degradation in enzymes including central metabolic pathways has been reported for *St. aureus* after PDI with exogenous added derivatives of porphyrins [28], for *E. coli* PDI with Zn-porphyrin [25] and for some other bacteria [20].

The key enzymes in oxidative phosphorylation, F1F0 ATPase, are sensitive to PDI for dormant *Msm*, similar to *E. coli* for PDI mediated by meso-tetra(N-methyl-4-pyridyl)porphine [29]. This means that PDI causes damage to the global energy-producing system in dormant *Msm* cells. Other vitally important proteins sensitive to PDI include ribosomal proteins, numbers of which disappeared from the pool of intact proteins (Tables S1 and S2 and Figure 9).

Previously, we found that dormant mycobacteria are enriched by proteins and enzymes protecting cell structures from oxygen stress and prevent protein aggregation under different stressful conditions. Evidently, these systems play an important role in maintaining dormant cells’ integrity and intactness under non-replicative conditions [17]. According to the results obtained in this present study, some of these “defense proteins” exhibited sensitivity to PDI (chaperonin GroEL, trigger factor/M*SMEG_4674*, *MSMEG_3932* (alpha-crystallin), thioredoxin/M*SMEG_4917*, copper/zinc superoxide dismutase/M*SMEG_0835* (sodC), antioxidant AhpC/TSA family protein/M*SMEG_4753*, glyoxalase family protein/M*SMEG_5680*). This makes dormant cells highly vulnerable to harmful factors during their long stay in a non-replicative state. From the published results, after PDI of active bacterial cells with exogenous PSs, some proteins participating in protection are resistant to illumination (like SOD, GroEL) while some of them (e.g., chaperone DnaK) are sensitive [20,28].

According to Table S1, the majority of proteins in dormant *Msm* cells affected by PDI have membrane localization. Likely, this is due to the hydrophobic nature of coproporphyrin tetra methyl ester as a major component of the endogenous free porphyrin pool in dormant *Msm* cells [5]. Apart from membrane localization, photosensitivity of a particular protein may be determined by the spatial proximity of photosensitizers to essential amino acids or structural motifs. The overall enzyme structure and amino acid composition may also greatly influence the enzyme’s susceptibility to light-induced damage. The significance of membrane localization was proven by the estimation of sensitivity of membrane-bound respiratory chain activity to PDI (Figure 10). In these experiments, the activity of the initial part of the respiratory chain, including dehydrogenases, was monitored by DCPIP, a known electron acceptor interacting in the membrane with mobile menaquinone. PDI, indeed, causes substantial depression in DCPIP reduction, as well as the inhibition of activity of the whole respiratory chain (measured by the rate of oxygen consumption). Another electron acceptor tetrazolium salt revealed similar suppression of its reduction by a respiratory chain in *E. coli* treated by PDI in the presence of exogenous porphyrins [25]. High sensitivity of the respiratory chain to PDI makes it supposedly one of the main targets for PDI-controlled cell viability. These experiments were performed using stationary phase bacterial cells. Both the stationary phase of mycobacteria and the dormant state are characterized by a significant reduction in metabolic activity including respiratory chain activity in comparison with multiplying cells. Nevertheless, the stationary phase bacteria (72 h) reveal measurable respiration, which we used as a control in our experiments (Figure 10). Regardless of the fact that the respiratory chain in dormant mycobacteria displays negligible activity [17,30], a lot of proteins and enzymes are preserved in these forms of bacteria, including enzymes of the respiratory chain [17]. Therefore, the harmful effect of illumination would have an effect on dormant forms similar to the stationary phase cells. The destruction of respiratory chain proteins during light exposure can lead to a reduction of the ability of dormant mycobacteria to restore growth processes.

In conclusion, according to the present study, PDI mediated by endogenous porphyrins damages proteins (mainly membrane-bound) of dormant mycobacteria, which are key enzymes in vital metabolic pathways. Because of such multiple targeting, PDI action could be considered as an effective approach for killing dormant bacterial forms, taking into consideration the insensitivity of these forms for conventional antibiotics which inactivate vegetative bacteria. It is also important that PDI destroyed the synthesis of proteins (inactivation of ribosomal proteins) and nucleic acids (inactivation of DNA polymerase III and DNA-directed RNA polymerases), the processes which are necessary for dormant cell reactivation and their transition to multiplying bacteria.

Thus, PDI based on endogenous PSs could be a perspective approach to inactivate dormant mycobacteria and combat a latent form of mycobacteriosis, first of all with surface localization.

## 4. Materials and Methods

### 4.1. Formation of the Dormant Forms of M. smegmatis upon the Medium Self-Acidification

The strain *Mycobacterium smegmatis mc^2^155* was obtained from the culture collection All-Russian Collection of Microorganisms of G.K. Skryabin Institute of Biochemistry and Physiology of Microorganisms RAS. *M. smegmatis* cells were initially grown in a nutrient broth (NB) (Himedia, Mumbai, India) at 37 °C for 24 h, with shaking at 200 rpm. The inoculum (1 mL) was added to 100 mL of Sauton medium, containing (g L^−1^) K_2_HPO_4_, 0.5; MgSO_4_.7H_2_O, 1.4; L-Asparagine, 4.0; ferric ammonium citrate, 0.05; Na_3_ (citrate), 2.0; ZnSO_4_, 0.001 (Sigma Aldrich, St. Louis, MO, USA) and glycerol (Panreac, Castellar del Vallès, Spain), 60 mL. For dormant cell formation, the initial pH value was 6.0 (instead of the pH 7.0 that is usually used in the usual Sauton composition) [30]. To prevent aggregation of the cells, Tween 80 was added (0.05%). The culture was grown at 37 °C, while constantly being stirred (200 rpm) for 18 days until a constant pH value (ca 6.0) of the medium was established [30]. Dormant cells were stored for 5 months without agitation in 15 mL capped tubes in the dark. In some experiments, an elevated concentration of Mg^2+^ by 5 times (25 mM) and Zn^2+^ by 10 times (62 µM) was used to prepare the Sauton medium. These experiments were repeated ten times in three technical replicas.

In experiments for the estimation of respiratory chain activity, active cells of *Msm* were grown in a standard Sauton medium for 72 h in the presence of 3 mM 5-aminolevulinic acid. These experiments were repeated three times in three technical replicas.

### 4.2. Viability Estimation by MPN

The most probable number (MPN) assay was carried out in 48-well plastic plates filled with 0.9 mL special medium for the most effective reactivation of dormant *Msm* cells. This medium contains 3.25 g of nutrient broth (Himedia, India) dissolved in 1 L of a mixture of Sauton medium (0.5 g KH_2_PO_4_; 1.4 g MgSO_4_·7H_2_O; 4 g L-Asparagine; 0.05 g ferric ammonium citrate; 2 g sodium citrate; 0.01% (*w*/*v*) ZnSO_4_·7H_2_O per litre, pH 7.0), Middlebrook 7H9 liquid medium (Himedia, Mumbai, India) and RPMI (Thermo Fisher Scientific, St. Louis, MO, USA) (1:1:1) supplemented with 0.5% *v*/*v* glycerol, 0.05% *v*/*v* Tween 80 and 10% ADC (Himedia, Mumbai, India). Appropriate serial dilutions of the cells (100 μL) were added to each well. Plates were sealed and statically incubated at 37 °C for 14 days. The MPN values were calculated [31] based on the well pattern originating from the visible bacterial growth at the corresponding dilution point. These experiments were performed two times in three technical replicas.

### 4.3. Infection of Macrophages with Dormant M. smegmatis Forms

*M. smegmatis* phagocytosis by peritoneal macrophages in vitro.

Peritoneal macrophages were obtained as described previously [32]. Then, 50 × 10^3^ macrophages per well were incubated in 96-well flat-bottom plates in 100 µL RPMI 1640 supplemented with 5% FCS, 10 mM HEPES, 2 mM L-Glutamine for 1 h for adhesion. Then, mycobacteria *M. smegmatis* at MOI 1, 5 and 10 were added for 16 h. Then, wells were washed twice carefully with warm RPMI 1640 to remove residual extracellular bacteria and were used for illumination. The control wells were left unilluminated. These experiments were performed two times in three technical replicas.

### 4.4. Pigment Extraction from the Cells

The pigment was extracted from the biomass according to Bligh and Dyer [33]. One ml of chloroform and 2 mL of methanol were added to the wet biomass of the cells (0.8 g). Cells were agitated for 2 h in the extraction mixture with subsequent centrifugation (2000× *g*), followed by the addition of 1 mL of water and 1 mL of chloroform (to separate the phases). The chloroform layer was washed three times with 0.1 M NaCl and was further analyzed using HPLC (Aquilon, Styer, Podolsk, Russia).

The concentration of porphyrins in the obtained extracts was determined spectrophotometrically according to spectral absorption coefficients [34]. These experiments were performed four times in three technical replicas.

### 4.5. Spectrophotometric and Fluorescence Analysis of Porphyrin in Extracts

The absorption spectra of the extracts were recorded in a 1 cm cuvette on a spectrophotometer Cary-50. Excitation and fluorescence spectra were recorded in a 3 mm cuvette on a Cary Eclipse spectrofluorometer in fluorescence mode at room temperature. These experiments were performed two times in three technical replicas.

### 4.6. Porphyrin Estimation by HPLC

Chloroform layers of porphyrin extracts were analyzed by HPLC (Aquilon, Stayer, Podolsk, Russia) under the following parameters: registration wavelength, 400 nm; column C18 (Tosoh Bioscience, Tokyo, Japan); a gradient mode was used with Buffer A containing 0.1 M aqueous NaH_2_PO_4_ in CH_3_CN (28:15 *v*/*v*, pH 5.3) and Buffer B containing 0.1 M aqueous NaH_2_PO_4_ in CH_3_CN (60:130 *v*/*v*, pH 5.3).With flow rate, 1 mL min^−1^ and injection sample size 20 μL, the gradient program consisted of the following phases: 0–5 min 100% Buffer A (0% Buffer B); 5–10 min gradient from 100% Buffer A to 0% (0–100% B); 10–70 min 0% Buffer A (100% B). The spectrofluorometer was set at 400 nm for the absorption, the fluorescence excitation wavelength was 400 nm and fluorescence emissions were detected at wavelengths 580 and 620 nm. Coproporphyrin III, Zn-coproporphyrin III and coproporphyrin tetra methyl ester (Calbiochem, San Diego, CA, USA) were used as standards. These experiments were performed two times in three technical replicas.

### 4.7. Photodynamic Inactivation

Suspensions of dormant (5 mo.) *Msm* with OD (600 nm) = 0.2, which correspond to ca. 10^7^ bacteria per ml, were used for the light inactivation experiments. We pipetted 100 µL in the wells of a 96-well plate (Nunc). These samples were illuminated by LED SOLIS-4C (ThorLabs, Newton, NJ, USA), as specified below in Table 5.

Illumination was for 5, 15, or 30 min and the temperature of samples in the microplate wells during illumination times was measured with the thermo pare probe of a Fluke 287 multimeter (±0.2 °C). The temperature was lower than 40 °C in the wells during all the experiments.

After illumination of the samples, serial tenfold dilutions (10^−1^ to 10^−7^) were prepared in NB with 0.05% Tween-80 medium, and were inoculated in 48-well plastic plates for MPN determination. These experiments were performed five times in three technical replicas.

### 4.8. Sample Preparation for SDS Electrophoresis

Control (unilluminated) and illuminated dormant bacterial cells obtained in four biological replicates were pooled (total volume 200 µL for each replicate; the cell amount was ca. 70 mg wet weight) for 1D electrophoretic analysis. It is known that pooling allows us to obtain an average expression of a particular protein which matches the mean expression of that protein after averaging several individual replicates [35]. The pooling should reduce the influence of the technical factors (especially during the destruction of dormant cells and extraction of proteins) on the result of 1D electrophoresis. This approach has been previously used in mycobacterial proteomic studies [17,36]. Bacteria were harvested via centrifugation at 8000× *g* for 15 min and washed three times with a buffer containing 50 mM Tris-HCl, 20 mM MgCl_2_ (pH 7.4). The bacterial pellet was re-suspended in a lysis buffer (a buffer of the same composition with the addition of 2% SDS), incubated for 2 h with stirring on a shaker (pr-ma II 50 RPM) then disrupted with zirconium beads on a bead beater homogenizer (MP Biomedicals FastPrep-24) for 1 min, 10 times. The bacterial lysate was centrifuged at 16,000× *g* for 15 min at 4 °C. The concentration of proteins was measured in the lysates (to equalize the concentration of proteins during application).

### 4.9. Protein Amount Determination

600 µL of Pierce reagent (Thermo Fisher Scientific, San Diego, CA, USA) was added to 40 µL of the analyzed protein solution. The samples were incubated for 5 min at room temperature. The optical density was measured on a spectrophotometer at 660 nm.

The absorbance at 660 nm was determined and adjusted to the calibration curve of the bovine serum albumin. A corresponding buffer was used for control.

### 4.10. SDS Electrophoresis

Protein electrophoresis was performed in 12% PAAG under denaturing conditions in a standard Tris-glycine buffer in a vertical Mini-Protean Tetra electrophoresis chamber (Bio-Rad, Hercules, CA, USA). Protein markers were used as standards for electrophoresis (Thermo Fisher Scientific, San Diego, CA, USA).

The gels were stained with Coomassie CBBG-250 (Roti-Blue Carl Roth, Karlsruhe, Germany).

The gels’ images were captured using Syngene G:BOX Gel & Blot Imaging Systems (Syngene, Cambridge, UK). Gel images stained with Coomassie were analyzed using TotalLab TL120 software to calculate line density.

Each visible stained protein line (band) for unilluminated and illuminated samples, or samples obtained from dormant bacteria grown under a standard and elevated concentrations of metal ions, were excised manually from the gel and analyzed using MALDI-TOF. The MS/MS data obtained from the MALDI-TOF were subjected to a Mascot Protein Database (MSDB) search to identify proteins. Proteins with coverage < 10% were not considered further. Protein functional roles were obtained from the Mycobrowser database (https://mycobrowser.epfl.ch, accessed on 30 July 2023). Each sample for 1D analysis was performed in two technical replicates.

### 4.11. Protein Identification by MALDI-TOF

All fractions excised from 1D electrophoresis slab gels were hydrolyzed via trypsin digestion. The extracted tryptic peptides were analyzed by MALDI-TOF as described previously, with some modifications. A sample (0.5 μL) was mixed with the same volume of 20% (*v*/*v*) acetonitrile solution containing 0.1% (*v*/*v*) trifluoroacetic acid and 20 mg/mL 2,5-dihydroxybenzoic acid, and then air-dried. Mass spectra were obtained on a Reflex III MALDI-TOF mass spectrometer with a UV laser (336 nm) in a positive ion mode in the range of 500–8000 Da. Calibration was performed in accordance with the known peaks of trypsin autolysis.

For MS/MS analysis, the mass spectra of the fragments were recorded with a Bruker Ultraflex MALDI-TOF mass spectrometer in tandem mode (TOF-TOF) with detection of positive ions. The proteins were identified using Mascot software in Peptide Fingerprint mode (Matrix Science, Boston, MA, USA). The accuracy of the mass measurement MH+ was 0.01% (with a possibility of modifying cysteine by acrylamide and methionine oxidation).

### 4.12. Respiration, DCPIP Reduction

Endogenous respiratory chain activity (complex I) was determined via the reduction of DCPIP (2,6-dichlorophenolindophenol) in the presence of menadione monitored spectrophotometrically at 600 nm. The reaction mixture (4 mL) contained 0.2 μmol 2,6-DCPIP, 0.6 μmol menadione, and 400 μL of the cell suspension in a Sauton medium (pH 7.4).

The intensity of the respiratory chain functioning was assessed by the rate of total oxygen consumption using the Expert-009 oximeter (Econix Expert, Moscow, Russia). The reaction mixture (70 µL) contained 10 µL suspension of *M. smegmatis* cells in Sauton medium (OD_600nm_ 0.4), 1 µL of 5 mM NADH or 1 µL of 0.5 M malate in 20 mM Tris-HCl, 1 mM MgSO4 and pH 7.4 buffer. The analysis of oxygen consumption was processed using the Expert-00x program. These experiments were performed five times in three technical replicas.

## Figures and Tables

**Table 1:** Oxidoreductases of dormant *M. smegmatis* cells sensitive to light illumination.

Gene Name	Product	Time of Illuminations		
		0 min	5 min	15 min
*MSMEG_0777*	F420-dependent glucose-6-phosphate dehydrogenase	+	−	−
*MSMEG_1030*	(NAD(P)/FAD-dependent oxidoreductase	+	−	−
*MSMEG_3106*	quinone oxidoreductase	+	+	−
*MSMEG_3232*	cytochrome D ubiquinol oxidase subunit II	+	+	+
*MSMEG_3233*	cytochrome D ubiquinol oxidase subunit I	+	−	−
*MSMEG_4645*	alpha oxoglutarate ferredoxin oxidoreductase, beta subunit	+	−	−
*MSMEG_4935*	epsilon subunit ATP synthase F1-F0	+	+	+
*MSMEG_4936*	beta subunit ATP synthase F1-F0	+	−	−
*MSMEG_4937*	gamma subunit ATP synthase F1-F0	+	+	+

+ protein presence in sample, − protein absence in sample. 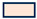 Proteins disappeared after 5 min of illumination. 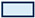 Proteins stable after 15 min of illumination. 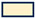 Proteins disappeared after 15 min of illumination.

**Table 2:** Various metabolic enzymes of dormant *M. smegmatis* cells sensitive to light illumination.

Gene Name	Product	Time of Illuminations		
		0 min	5 min	15 min
*MSMEG_0029*	anthranilate synthase component 2	+	−	−
*MSMEG_1342*	(3R)-hydroxyacyl-ACP dehydratase subunit HadC	+	−	−
*MSMEG_3564*	bacterioferritin	+	+	−
*MSMEG_3767*	acyl-CoA synthetase	+	−	−
*MSMEG_4340*	NAD/mycothiol-dependent formaldehyde dehydrogenase	+	−	−
*MSMEG_5136*	pyridoxine 5’-phosphate (PNP) oxidase	+	−	−
*MSMEG_5191*	siderophore binding protein	+	+	−
*MSMEG_5243*	pyridoxine 5’-phosphate (PNP) oxidase	+	−	−
*MSMEG_5471*	UTP--glucose-1-phosphate uridylyltransferase	+	−	−
*MSMEG_5485*	molybdopterin biosynthesis protein	+	+	−
*MSMEG_5702*	molybdenum cofactor synthesis domain protein	+	−	−
*MSMEG_5824*	phosphoribosylformylglycinamidine synthase II	+	−	−
*MSMEG_5892*	trehalose-6-phosphate synthase	+	−	−
*MSMEG_6392*	polyketide synthase	+	−	−
*MSMEG_6520*	orotate phosphoribosyltransferase	+	−	−

+ protein presence in sample, − protein absence in sample. 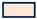 Proteins disappeared after 5 min of illumination. 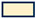 Proteins disappeared after 15 min of illumination.

**Table 3:** Regulatory proteins of dormant *M. smegmatis* cells sensitive to light illumination.

Gene Name	Product	Time of Illuminations		
		0 min	5 min	15 min
*MSMEG_0937*	DNA-binding response regulator RegX3	+	−	−
*MSMEG_0994*	regulators DNA-binding response regulator ResD	+	−	−
*MSMEG_3246*	sensor part of a two-component regulatory system	+	−	−
*MSMEG_3647*	glycogen accumulation regulator GarA	+	−	−
*MSMEG_5488*	the DNA-binding response regulator	+	+	−
*MSMEG_6091*	negative regulator of genetic competence ClpC/mecB	+	−	−
*MSMEG_6896*	the single-stranded DNA-binding protein	+	+	−

+ protein presence in sample, − protein absence in sample. 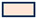 Proteins disappeared after 5 min of illumination. 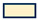 Proteins disappeared after 15 min of illumination.

**Table 4:** Enzymes of defense systems of dormant *M. smegmatis* cells sensitive to light illumination.

Gene Name	Product	Time of Illuminations		
		0 min	5 min	15 min
*MSMEG_0835*	copper/zinc superoxide dismutase	+	−	−
*MSMEG_0880*	chaperonin GroEL	+	−	−
*MSMEG_3932*	alpha-crystallin stress protein	+	−	−
*MSMEG_4674*	trigger factor	+	−	−
*MSMEG_4753*	antioxidant protein	+	−	−
*MSMEG_4917*	thioredoxin	+	−	−
*MSMEG_5680*	glyoxalase family protein	+	−	−

+ protein presence in sample, − protein absence in sample. 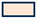 Proteins disappeared after 5 min of illumination.

**Table 5:** Wavelength, power density, light doses and time of illumination of *Msm* suspensions.

LED	Power Density (mW/cm^2^)	Time of Illuminations (min) and Light Doses (J/cm^2^)			
565 nm	180	5 min	15 min	30 min	60 min
		54 J/cm^2^	162 J/cm^2^	324 J/cm^2^	648 J/cm^2^

## Data Availability

Data is contained within the article or Supplementary Materials.

## Supplementary Materials

The following supporting information can be downloaded at: https://www.mdpi.com/article/10.3390/ijms241813968/s1.
